# Manual on the proper use of sodium astatide ([^211^At]NaAt) injections in clinical trials for targeted alpha therapy (1st edition)

**DOI:** 10.1007/s12149-021-01619-2

**Published:** 2021-05-12

**Authors:** Tadashi Watabe, Makoto Hosono, Seigo Kinuya, Takahiro Yamada, Sachiko Yanagida, Masao Namba, Yoshihide Nakamura

**Affiliations:** 1 Department of Nuclear Medicine and Tracer Kinetics, Graduate School of Medicine, Osaka University, 2-2 Yamada-oka, Suita, Osaka 565-0871 Japan; 2 Department of Radiology, Faculty of Medicine, Kindai University, 377-2 Ohno-Higashi, Osaka-Sayama, Osaka, 589-8511 Japan; 3 Japanese Society of Nuclear Medicine, 3-1-17 Nishi-Azabu, Minato-ku, Tokyo, 106-0031 Japan; 4 Atomic Energy Research Institute, Kindai University, Higashi-Osaka, 3-4-1 Kowakae, Osaka, 577-8502 Japan; 5 Japan Radioisotope Association, 2-28-45 Honkomagome, Bunkyo-ku, Tokyo, 113-0021 Japan; 6 Chiyoda Technol Corporation, 1-7-12 Yushima, Bunkyo-ku, Tokyo, 113-8681 Japan

**Keywords:** [^211^At] sodium astatide, Targeted alpha therapy, Radiation protection, Thyroid cancer

## Abstract

We present the guideline for use of [^211^At] sodium astatide (NaAt) for targeted alpha therapy in clinical trials on the basis of radiation safety issues in Japan. This guideline was prepared by a study supported by the Ministry of Health, Labour, and Welfare, and approved by the Japanese Society of Nuclear Medicine on 8th Feb, 2021. The study showed that patients receiving [^211^At]NaAt do not need to be admitted to a radiotherapy room and outpatient treatment is possible. The radiation exposure from the patient is within the safety standards of the ICRP and IAEA recommendations for the general public and caregivers. Precautions for patients and their families, safety management associated with the use of [^211^At]NaAt, education and training, and disposal of medical radioactive contaminants are also included in this guideline. Treatment using [^211^At]NaAt in Japan should be carried out according to this guideline. Although this guideline is applied in Japan, the issues for radiation protection and evaluation methodology shown here are considered internationally useful as well.

## Purpose of this safety management manual

This safety management manual is a compilation of implementation guidelines for clinical trials with the purpose of ensuring the safe handling of [^211^At] sodium astatide ([^211^At]NaAt) in compliance with the principles of the Safety Guidelines for the “release of patients administered radiopharmaceuticals” [1, 2], issued by the Ministry of Health, Labour and Welfare, Japan on the application of sodium astatide ([^211^At]NaAt) injections in the treatment of differentiated thyroid cancer.

Currently, radioactive iodine (^131^I-NaI) is used in nuclear medicine to treat differentiated thyroid cancer. However, despite repeated administration, favorable results are not obtained in all patients. Particularly, in some patients, the treatment effects are not enough for metastatic lesions regardless of whether they take ^131^I-NaI [3]. As such, favorable outcome are expected for a treatment with alpha nuclides, which have greater therapeutic effects. As alpha rays deposit a large amount of energy over short-range that covers several cells, greater therapeutic effects can be obtained while limiting effects on the surrounding cells by selectively targeting cancer cells.

Furthermore, the use of ^131^I-NaI in patients with multiple metastases requires isolated hospitalization in a dedicated radiotherapy room. Confinement in this room imposes a heavy mental and physical burden on patients, and many medical institutions cannot afford to maintain such a room due to its high cost. Existing studies have shown that outpatient treatment is possible with astatine (^211^At), which is a short-range alpha nuclide [4].

Therefore, healthcare professionals who perform [^211^At]NaAt treatment must sufficiently understand the physical properties of ^211^At and the chemical properties of [^211^At]NaAt, to provide proper explanation and guidance to patients.

One of the features of nuclear medicine is molecular targeted therapy, wherein the administered radiopharmaceutical is selectively accumulated in the lesion (e.g., metastatic tumor cells throughout the patient’s body), and localized irradiation is performed. Measures for safe handling of [^211^At]NaAt, and prevention of radiation exposure and contamination are essential in nuclear medicine to ensure the safe administration of minimally invasive and patient-friendly treatments. Therefore, patients, family members, and other concerned parties must fully understand the aspects of nuclear medicine treatment.

This manual incorporates the points established in the Medical Care Act, and recommendations on radiological protection established by international organizations [4–8]; ideally, healthcare facilities that implement this treatment must follow the requirements covered in this manual to ensure radiation safety. The following points are summarized in this manual (safety management edition):

1. Guidelines on facility management.
2. Radiation exposure protection.
3. Storage and disposal of medical radioactive contaminants.

Furthermore, in the process of carrying out this treatment, the following conditions concerning the standards of the facility must be satisfied:

1. Healthcare facilities implementing this treatment must comply with the standards on protection from medical radiations stipulated in relevant laws and complete legal procedures.
2. This treatment should be carried out at healthcare facilities with full-time doctors and radiology technologists with sufficient knowledge and experience in handling radiopharmaceuticals. Moreover, it should be performed in hospitals with specialized knowledge and experience in the treatment of thyroid cancer.

## Organizational initiatives in healthcare facilities that use [^211^At]NaAt

Considering the unique characteristics of this drug, hospitals that perform this treatment must meet the requirements stipulated here, namely to perform this treatment with medical teams consisting of physicians, radiology technologists involved in radiological safety management, nurses involved in patient care and assistance, and other health professionals.

## Structure and safety management in healthcare facilities

As stipulated in Articles 30–8, 30–9, and 30–11 of the Medical Care Act Implementing Regulations, all rooms of healthcare facilities that perform this treatment should have structural facilities that comply with the standards stipulated in Articles 30–13 to 30–26. The facilities must be approved by the prefectural governor who has jurisdiction over them. For managers of healthcare facilities that perform this treatment to ensure the safety of medical care, safe handling of [^211^At]NaAt, and radiological safety, as stipulated in Article 1, item 3–2 of the Medical Care Act Implementing Regulations, there is a need to establish a safety management system for all healthcare personnel involved in administering this treatment.

## Assignment and role of a radiological safety management supervisor and managers

Managers of healthcare facilities that perform this treatment must assign a radiological safety management supervisor. Radiological safety management supervisors shall oversee treatment at the facility and manage the implementation of education and trainings for the healthcare professionals involved.

Furthermore, should the supervisor specified in Article 1–11, clause 2, Item 3–2 of the Medical Care Act Implementing Regulations (medical radiological safety manager) serve concurrently as the radiological safety supervisor specified in this manual, they shall not be reassigned. Should different person be assigned to each post, the relationship between them must be clarified and a system must be established so that the treatment can be performed.

The supervisors and investigators in healthcare facilities that perform this treatment must assign one or more radiological safety managers depending on the status of the health facility. Radiological safety managers shall perform duties related to ensuring and managing radiological safety of the treatment and shall be involved in the implementation of related education and training, under the supervision of the radiological safety management supervisor.

To implement this treatment in accordance with this manual, the following conditions must be met:

1. This drug should be administered to patients with differentiated thyroid cancer.
2. Details of the treatment must be presented to the patient and/or family (or caregiver) by a specialist, and the patient and/or family (or caregiver) must provide informed consent.
3. Appropriate sewage and toilet facilities must be available in the patient’s residence after discharge.
4. Patient must be able to lead a life in which they can make independent judgments and actions.
5. Minimal contact between the patient and children or pregnant women must be ensured after discharge.

## Characteristics of ^211^At and [^211^At]NaAt

The physical properties of ^211^At as a radionuclide are shown in Table 1. ^211^At undergoes alpha decay to ^207^Bi (41.8%) and electron capture decay to ^211^Po (58.2%) with a physical half-life of 7.214 h. The daughter nuclide of ^211^Po decays to stable ^207^Pb by alpha rays emissions with a half-life of 0.516 s, resulting 100% alpha rays emission during the disintegration of ^211^At. This radionuclide is produced by the ^209^Bi (α, 2n) ^211^At reaction. At is a halogen with an atomic number of 85. As a halogen, the pharmacokinetics of At are similar to those of iodine. It is reported that At has several chemical forms such as At^−^, At^+^, At(OH)_2_^−^, AtO_2_^−^, AtO(OH)^2−^, and AtO^+^. The At^−^ (monovalent anion) contained in this drug is distributed to the stomach, thyroid, salivary glands, testis, and so forth, via a sodium-iodide symporter, and it is excreted in urine [9]. The biodistribution of [^211^At]NaAt, 1, 3, 6, and 24 h after intravenous administration in normal mice, was evaluated by measuring the radiation dose and weight of each dissected organ. Furthermore, the residence time (h) was calculated from the area under the time-activity curve. Assuming that biodistribution in the human body is the same as in mice, the residence time was entered into the internal exposure dose calculation software IDAC-Dose v2.1, and the absorbed dose (mGy/MBq) of each organ was estimated (Table 2) [10].

**Table 1:** Physical properties of ^211^At

Half-life	Decay mode	Maximum alpha energy (MeV) and emission rate	Main photon energy (MeV) and emission rate	Emission of internal conversion electrons per 100 disintegrations (%)	Effective dose rate constant (μSv・m^2^・MBq^−1^・h^−1^)
7.214 h Daughter ^207^Bi ^*211^Po	α EC	5.867–41.8% Others 58.20%	0.670–0.0035% 0.743–9.5 × 10^–4^% 0.687–0.26% 0.0787–31.1% Po-K_α_ 0.0906–8.5% Po-K_β_ 0.0124–18.9% Po-L	0.015	0.00580 0.00644^*^

*Includes contribution from ^211^Po, which is in radioactive equilibrium

[Source: Radioisotope Pocket Data Book (12th Edition) published by the Japan Radioisotope Association, 2020]

**Table 2:** Estimated absorbed dose after [^211^At]NaAt administration in adult humans

Organ/tissue	Absorbed dose (mGy/MBq)
Brain	0.0108
Thyroid gland	19.1
Salivary glands	3.43
Myocardium	0.150
Lungs	0.0264
Liver	0.145
Stomach	4.79
Small intestine	0.352
Colon	0.199
Kidneys	0.417
Pancreas	0.219
Spleen	0.433
Testes	2.01
Urinary bladder	0.459
Red bone marrow	0.0901

## Criteria for release of patients administered radiopharmaceuticals

The criteria for release (PSD No. 70 Notice) were issued as a guideline for ensuring the quality of life of treated patients, and the safety of the general public and caregivers from radiation. This was notified as an interpretation of the proviso stipulated in Article 30–15, clause 1 of the Medical Care Act Implementing Regulations. The outline of the release criteria is as follows:

1. Scope of application: When a patient to whom a radiopharmaceutical was administered is released or returns home from a medical radioisotope room or radiotherapy room inside a hospital.
2. Release criteria: The criteria for dose limit is 1 mSv per year for the general public and 5 mSv per case for the caregiver, respectively, taking into consideration the mutual benefits for the patient and caregiver.
  Specifically, release or returning home is permitted if any of the following (1 to 3) apply.
  - Release criteria based on dose.
    Release is permitted provided that the administered dose or residual radioactivity in the body does not exceed the values shown in Table 3.
  - Release criteria based on measured dose rate.
    Release or returning home is permitted provided that the dose rate measured at a distance of 1 m from the patient’s body surface does not exceed the values in Table 4.
  - Release criteria based on cumulative dose calculation for each patient.
    Based on this, release or returning home is permitted in the following cases (Table 5).
3. Release record.
  Once release is permitted, the following must be recorded and stored for two years after release:
  - Administered dose, release date and time, and dose rate at release.
  - Notes and guidance for mothers with nursing infants.
  - If release is permitted based on (c) in 2) of the preceding paragraph, the calculation method of the cumulative dose that permitted release.

**Table 3:** Radioactivity level for release and returning home of patients administered radiopharmaceuticals

Radionuclides for medical use	Administered dose or residual radioactivity in the body (MBq)
Strontium (^89^Sr)	200^*^^1^
Iodine (^131^I)	500^*^^2^
Yttrium (^90^Y)	1,184^*^^1^

^*1^ Maximum administered dose

^*2^ The radioactivity of ^131^I is derived from the dose by adding the internal exposure due to the inhalation of ^131^I released with the patient’s expiration to the external exposure dose from the patient’s body

**Table 4:** Dose rate for release and returning home of patients administered radiopharmaceuticals

Radionuclides for medical use	1-cm equivalent dose rate at 1 m from the patient’s body surface (μSv/h)
^131^I	30

**Table 5:** Cases that meet the release criteria based on the cumulative dose evaluation for each patient

Radionuclides for medical use	Scope of application	Dose (MBq)
Iodine (^131^I)	Residual thyroid ablation after total thyroidectomy in differentiated thyroid cancer without distant metastasis^a^	1110
Radium (^223^Ra)	Treatment of castration-resistant prostate cancer with bone metastasis^b^	12.1^c^

^a^ Limited to implementation in accordance with the guidelines prepared by related academic societies (“Outpatient treatment with I-131 (1,110MBq) for the purpose of residual thyroid destruction”).

^b^ Limited when it is performed by administering 55 kBq/kg per dose of radium chloride (Ra-223) injection at 4-week intervals (up to 6 times) according to the implementation guidelines prepared by related academic societies (“Proper use manual for internal therapy using radium chloride (Ra-223) injection”).

^c^ Maximum dose

If release or returning home has been permitted, precautions and guidance on daily life must be explained in writing or verbally to avoid unnecessary exposure of third parties. If the patient has a nursing infant, proper explanation must be provided, and due circumspection and guidance must be applied. Regarding protection with respect to the physical characteristics of radionuclides, explanation to patients and caregivers, and other safety management issues, refer to the guidelines prepared by radiology-related academic societies.

The duration of contact with the patient, distance from the patient, and radiation dose are factors related to the external exposure dose. Therefore, the exposure coefficient^*^, a factor to be considered when evaluating the exposure dose of a third party, is set according to the third party’s relationship with the patient. Exposure coefficient is defined as the ratio of the cumulative radiation dose assumed to be received by third parties to the cumulative radiation dose when at a distance of 1 m from the point source (patient) of the nuclide for an infinite time (time until all nuclides are fully decayed).

1. Exposure coefficient for caregivers: 0.5
  Previous report stated that 0.5 is an appropriate exposure coefficient for caregivers of patients administered radiopharmaceuticals requiring strict nursing care [12]. Furthermore, the same value was used as the exposure coefficient in a study, in Japan, which measured the exposure dose emitted from the patient [13]. Based on the results of the study, 0.5 was adopted as the exposure coefficient in the dose evaluation of caregivers after release or returning home.
2. Exposure coefficient for the general public: 0.25
  Previous study [12] reported 0.25 as a valid exposure coefficient based on the actual measured exposure dose of the patient’s family in an ordinary household. Hence, 0.25 was adopted as the exposure coefficient for family members, excluding caregivers, and the general public after release or returning home.

## Exposure dose of a third party from patient administered [^211^At]NaAt

The exposure dose of third parties (e.g., caregivers and the general public) includes both the external exposure because of radiation emitted from radioactive substances in the patient administered [^211^At]NaAt and the internal exposure owing to contamination with the patient’s excrement. The following is a combined evaluation of the doses to which third parties are exposed.

1. Evaluation of the external exposure dose
  Effective dose rate of external exposure is calculated at 1 m from patients administered [^211^At]NaAt as follows.
  Formula for calculating the dose rate of external exposure to a third party from a patient administered [^211^At]NaAt:
  $$I\, = \,A\, \times \,{\text{C}}\, \times \,F_{{\text{a}}} /L^{{2}} .$$
  where *I* is the effective dose rate at the calculated evaluation point [μSv/h], *A* is the residual radioactivity in the body of the treated patient [MBq], C is the effective dose rate constant of ^211^At [μSv・m^2^・MBq^−1^・h^−1^] (values in Table 1, 0.00644 [μSv・m^2^・MBq^−1^・h^−1^] is used),*F*_a_ is the effective dose transmission rate (if there are multiple barriers, the product of transmissions of each shield is the total transmission), and *L*is the distance from the radiation source to the evaluation point [m].
  Next, cumulative exposure dose of a third party was calculated from the patient administered [^211^At]NaAt as follows:
  Formula for calculating the cumulative effective dose if third party is continuously exposed to a patient administered [^211^At]NaAt:
  $$E = A \times \smallint_{0}^{\infty } \left( \frac{1}{2} \right)^{{\frac{t}{{\text{T}}}}} dt \times {\text{C}} \times {\text{f}}_{0}$$
  where *E* is the cumulative effective dose to which a third party is exposed [μSv], *A* is the residual radioactivity in the body of patient [MBq], C is the effective dose rate constant of ^211^At [μSv・m^2^・MBq^−1^・h^−1^] (values in Table 1, 0.00644 [μSv・m^2^・MBq^−1^・h^−1^] is used), Tis the physical half-life of ^211^At, and f_0_ is the exposure coefficient;*caregiver: 0.5, non-caregiver (general public): 0.25.
  Following factors are considered for the evaluation of cumulative exposure doses to caregivers and the general public from patients administered [^211^At]NaAt:
  - The cumulative dose to which a third party is exposed after release or returning home of a patient administered [^211^At]NaAt is evaluated using the effective dose rate at a distance of 1 m from the patient’s body surface.
  - The actual state of radioactivity in a patient administered [^211^At]NaAt depends on the physical half-life of ^211^At and the effective half-life of [^211^At]NaAt. However, for the safety evaluation, only the physical half-life is considered, assuming that [^211^At]NaAt is not excreted from the body.
  - The changes in the radioactivity of ^211^At inside the body will be estimated based on (a) and (b); summary of factors used to estimate the cumulative dose to which a third party is exposed to a patient administered [^211^At]NaAt:
    (c) The changes in the radioactivity of ^211^At inside the body will be estimated based on (a) and (b); summary of factors used to estimate the cumulative dose to which a third party is exposed to a patient administered [^211^At]NaAt:
  Maximum dose of [^211^At]NaAt: 1.0 GBq.
  Physical half-life of ^211^At: 7.214 h.
  Estimation of the cumulative dose is calculated for a third party who is exposed from the patient administered [^211^At]NaAt. The calculation of the effective dose rate of external exposure at a distance of 1 m from the patient’s body surface for a certain period of time after the administration of [^211^At]NaAt is as follows:
  The effective dose rate coefficient from Table 1 is 0.00644 [μSv⋅m^2^⋅MBq^−1^⋅h^−1^].
  The effective dose rate at a distance of 1 m from the body surface of the patient immediately after the administration of [^211^At]NaAt at the maximum dose of 1.0 [GBq] (1000 [MBq]) can be expressed as:
  $${\text{1}}000\left[ {{\text{MBq}}} \right] \times 0.00{\text{644}}\left[ {\mu {\text{Sv}} \cdot {\text{m}}^{{\text{2}}} \cdot {\text{MBq}}^{{ - {\text{1}}}} \cdot {\text{h}}^{{ - {\text{1}}}} } \right] \times 1\left[ {{\text{m}}^{{ - {\text{2}}}} } \right] = {\text{6}}.{\text{44}}\left[ {\mu {\text{Sv}} \cdot {\text{h}}^{{ - {\text{1}}}} } \right]$$
  Furthermore, since the area under the curve to infinity of the decay curve is calculated using half-life [h]/log 2, the cumulative dose until the effective dose rate decays to infinity at a distance of 1 m from the patient’s body surface is calculated as:
  $${6}.{44 }[\mu {\text{Sv}} \cdot {\text{h}}^{{ - {1}}} ] \times {7}.{214}/0.{6931 }\left[ {\text{h}} \right] = {67}.0{3 }\left[ {\mu {\text{Sv}}} \right]$$
  However, there is no shielding effect by the patient’s body that is the effective dose transmission is assumed to be 1.
  Considering the exposure factor, the cumulative doses for caregivers and the general public will be given as:
  ・Cumulative dose for caregiver (exposure coefficient = 0.5):
  67.03 μSv/case × 0.5 = 33.52 μSv/case
  ・Cumulative dose for general public(exposure coefficient = 0.25):
  67.03 μSv/case × 0.25 = 16.76 μSv/case
  In both cases, they are far below the dose limit for caregiver (5 mSv), dose limit for the general public (1 mSv).
2. Evaluation of the internal exposure dose
  Excrements of patients treated with [^211^At]NaAt may flow out into rivers through sewage, mainly in the form of urine, and may be used as drinking water after reprocessing. Therefore, in estimating the internal exposure dose, evaluation is needed assuming all the radioactivity administered to the patient will flow out into the river.
  As with the examination of the release criteria, the general public exposure dose will be examined using the Yodo River water system model in the Osaka area, where the utilization rate of purified treated water is high [12]. Regarding the exposure dose of caregivers, since astatine is a homologous to iodine, the exposure dose to caregivers is estimated with reference to the “Evaluation of dose received by caregivers from patients administered iodine-131” [12]. The results of these studies establish that it satisfies the annual general public dose limit of 1 mSv and the dose constraint per caregiver of 5 mSv as stated in the recommendations of the International Commission on Radiological Protection (ICRP) and the safety standards of the International Atomic Energy Agency (IAEA).
  Estimation of general public exposure dose is performed as follows. Of approximately 3000 cases of ^131^I-NaI treatment (as of 2010), it was assumed that treatment with [^211^At]NaAt would be performed in 10% (300 cases) of intractable cases [14]. If 10 [MBq/kg] of At is administered to a patient weighing 70 [kg], the total dose would be 700 [MBq/person], and considering the total annual usage in the Osaka area, it would be 0.70 × 300 × 0.102 = 21.4 [GBq/year] [12].
  To conduct safe evaluation, assuming that all ^211^At administered to the patient flowed into the Yodo River system, the radioactivity concentration in the Yodo River system would be 21.4 [GBq/year]/4.1 [TL/year] = 0.0052 [Bq/L]. Here,the annual average release into the Yodo River system (annual average from 1991 to 1995) is 4.1 [TL].
  The annual ^211^At intake of the general public (assuming use of 2 L of drinking water per day) would be 0.0052 [Bq/L] × 2 [L/d] × 365 [d/year] = 3.8 [Bq/year]. In this case, the annual internal exposure dose would be 3.8 [Bq/year] × 1.1 × 10^–5^ [mSv/Bq] = 0.04 [μSv/year]. Here, 1.1 × 10^–5^ [mSv/Bq] is the effective dose coefficient by oral ingestion of ^211^At [15].
  The value, 0.04 [μSv], is far below the general public annual dose limit of 1 [mSv] (1,000 [μSv]).
  Estimation of the exposure dose to caregivers is performed with reference to the previous report [12]. In a report [16] examining air contamination due to expired gas from the patients administered iodine-131, the maximum volatility of iodine, which is 1.4 × 10^–5^ per hour, was applicable to astatine. Furthermore, estimation is performed supposing that the volume of the room housing the patient is 30 [m^3^], the frequency of ventilation is once every hour, the daily respiratory volume of the care giver is 20 [m^3^], and the caregiver is constantly in the same room with the patient [12].
  The radioactivity ingested by the caregiver per 1 MBq of dose could be calculated as: 1 [MBq] × 1.4 × 10^–5^ [h^−1^] × 1/30 [m^−3^] × 1 [h] × 20 [m^3^/d] × 1/24 [d/h] × 10.41 [h] = 4.05 × 10^–6^ [MBq]. Here, the average life of ^211^At is 10.41[h].
  The effective dose of internal exposure associated with inhalation per 1 MBq (exposure coefficient = 0.5 [12]) is 4.05 × 10^–6^ [MBq] × 10^6^ [Bq/MBq] × 2.7 × 10^–5^ [mSv/Bq] × 0.5 = 5.47 × 10^–5^ [mSv] = 0.0547 [μSv], assuming that 700 [MBq] is administered to the patient per treatment, the internal exposure due to inhalation by the caregiver would be 0.0547 × 700 = 38.29 [μSv].
  Here, 2.7 × 10^–5^ [mSv/Bq] is the effective dose coefficient for inhalation of ^211^At [15].
  Adding the internal exposure due to general public ingestion, the internal exposure to the caregiver would give: 38.29 + 0.04 = 38.33 [μSv].
  This value is far below the dose limit for caregiver of 5 [mSv] (5000 [μSv]) per case.
3. Comprehensive evaluation of the external and internal exposure doses
  The results of a comprehensive evaluation of the external and internal exposure doses to which caregivers or the general public are exposed are shown below.
  Caregiver 33.52 [μSv] + 38.33 [μSv] = 0.072 [mSv].
  General public 16.76 [μSv] + 0.04 [μSv] = 0.017 [mSv].
  The exposure dose to caregivers and the general public is estimated to be 0.072 [mSv] and 0.017 [mSv], respectively. Both values meet the criteria for the recommended dose limit in each category.
  This is assuming exposure to a dose of 10 [MBq/kg] (once a year); however, the annual dose may increase depending on future clinical trial protocols. In that case, the maximum dose must be recalculated; however, as the estimated exposure doses to both the general public and caregiver are far below the dose limit, no significant effects can be expected.

## Release of patients administered [^211^At]NaAt

Cases where a patient who has just been administered [^211^At]NaAt is released from the medical radioisotope or radiotherapy room, may be able to satisfy the safety standards of the ICRP and IAEA recommendations, and the intention of the release criteria stated in the PSD No. 70 Notice.

Therefore, patients receiving [^211^At]NaAt do not need to be admitted to a radiotherapy room as stipulated in Article 30–15 of Medical Care Act Implementing Regulations.

## Precautions for patients and their families

After the administration of [^211^At]NaAt, trace amounts of radioactivity are present in body fluids (mainly blood), urine, and feces. Particularly, since most of the drug unabsorbed by the tumor is excreted through the urinary system, the precautions listed below should be documented and explained to patients and their family members before administration.

1. Precautions to be taken 2 days post-administration
  - If the patient bleeds, wipe off the blood with toilet paper and flush it down the toilet.
  - Wear disposable rubber gloves if the caregiver may possibly touch the patient’s urine, feces, or clothing contaminated by ^211^At.
  - If one’s hands or skin come into contact with patient’s blood or other bodily fluids, immediately wash the area with soap.
  - No sexual intercourse.
  - People living with the patient should be kept as far away as possible. A distance of 1 or 2 m should be kept from the patient when contact time is long. Particularly, minimize contact with children and pregnant women.
  - Avoid sleeping in the same bed as others. Sleep at least 2 m away or in a separate room if possible.
  - Patients should bathe after everyone else. After bathing, detergent should be used to thoroughly scrub the bathtub.
  - Avoid going to public places, including public transportation centers, supermarkets, shopping centers, movie theaters, restaurants, and sports centers.
  - Patient’s clothes should be washed separately; avoid mixing them with others’ clothes. Furthermore, sheets and underwear contaminated with blood or urine should be thoroughly pre-washed.
  - Precautions for urination, defecation, or vomiting
    Male patients should urinate while sitting.
    If feces or urine spills on the toilet bowl or floor, wipe it clean with toilet paper and flush it down the toilet.
    Rinse the toilet bowl twice after use.
    Wash hands thoroughly with soap after urination and/or defecation.
    Wash hands and skin thoroughly with soap if they come in contact with patient’s body fluids, excrement, or vomit.
2. Precautions for 1 week post-administration
  - Female patients should not breastfeed.
  - When using facilities where radiation detection is performed such as borders and airports, carry a medical certificate of the treatment.
3. Precautions for 3 months post-administration
  Patients administered [^211^At]NaAt should have contraception for 6 months post-administration, regardless of gender.
4. Radiation safety management for patients using diapers and urinary catheters
  For patients using diapers and urinary catheters, the following precautions should be taken early after administration (about 2 days). When handling diapers, urinary catheters, and urine storage bags, wear disposable gloves as a precaution for biohazard prevention.
  [Precautions when using diapers, urinary catheters, etc. (in-home or in-hospital)].
  - Vinyl sheets are recommended for patients with urinary incontinence using diapers.
5. If the patient uses the same urinary catheter after release from the radiotherapy room, discard the contents of the urine bag into the toilet, flush twice, and wash hands thoroughly.
6. For in-patients, replace the urinary catheter and urine bag before discharge.
7. Seal used diapers in a plastic bag and keep for 3 days after administration of [^211^At]NaAt.

[Precautions for disposing of diapers, urinary catheters, etc.]

- The diapers of treated patients used at home should be sealed in a plastic bag, so that the contents do not leak and treated as general waste. If necessary, dispose of them in accordance with the disposal method mandated by the local government.
- When disposing of infectious waste, such as diapers in the hospital, refer to “Handling Diapers of Radiopharmaceutical-administered Patients (Guidelines for Medical Professionals Practicing Nuclear Medicine) [17].”

## Regulatory laws governing clinical trials on [^211^At]NaAt

The regulatory laws regarding the prevention of radiation hazards when conducting clinical trials on [^211^At]NaAt are as follows:

1. Act Regulating Radioactive Isotopes: Nuclear Regulation Authority [18].
2. Medical Care Act [19] (Implementing regulations [20]): Ministry of Health, Labour and Welfare.
3. Act on Securing the Quality, Efficacy, and Safety of Products Including Pharmaceuticals and Medical Devices: Ministry of Health, Labour and Welfare.
4. Medical Practitioners’ Act: Ministry of Health, Labour and Welfare of Japan.
5. Pharmacists Act: Ministry of Health, Labour and Welfare of Japan.
6. Act for Medical Radiology Technicians: Ministry of Health, Labour and Welfare of Japan.
7. Industrial Safety and Health Act (Regulation on Prevention of Ionizing Radiation Hazards [21] and Working Environment Measurement Act): Ministry of Health, Labour and Welfare of Japan.
8. National Civil Service Act (Rules of the National Personnel Authority 10–5 [22]): National Personnel Authority.

## Standards for medical radioisotope rooms

Healthcare facilities that perform the treatment using [^211^At]NaAt must have medical radioisotope rooms, storage, and disposal facilities in compliance with the standards for radiation hazard prevention stipulated in Articles 30–8, 30–9, and 30–11 of the Medical Care Act Implementing Regulations.

## Standards for concentration limits in medical radioisotope rooms

Healthcare facilities that provide nuclear medicine treatments must meet the standards for concentration limits shown in Table 6 for the structural facilities of each room.

**Table 6:** Standards for dose and concentration limits for medical radioisotope rooms

Rooms	Medical Care Act
Rooms	Medical radioisotope rooms
	Storage facilities
	Disposal facilities
	Radiotherapy rooms
Dose and concentration limits in controlled areas	・Effective dose of external radiation: 1.3 mSv per 3 months ・Concentration of radioisotope (RI) in the air: The average concentration in 3 months is 1/10 of the concentration limit of the RI in the air ・Surface density of substances contaminated by RI: 1/10 of the surface density limit (alpha ray-emitting RI: 0.4 Bq/cm^2^, non-alpha ray-emitting RI: 4 Bq/cm^2^)
Dose and concentration limits in regular thoroughfares in RI facilities	・Effective dose outside the wall: 1 mSv per week ・RI concentration in air: the average weekly concentration is the RI concentration limit in air ・Surface density of substances contaminated by RI: Area density limit (alpha ray-emitting RI: 4 Bq/cm^2^, non-alpha ray-emitting RI: 40 Bq/cm^2^)
Dose standards at the boundaries of hospitals (including residential areas inside the hospital)	Effective dose: 250 μSv per 3 months
Exposure dose of in-patients	Effective dose should not exceed 1.3 mSv per 3 months

## Restrictions on places of use

Medical radioisotopes must be handled in a medical radioisotope room. However, this limitation does not cover temporary use in the operating room after taking appropriate protective and anti-contamination measures, radiotherapy room for patients who are difficult to transfer, or temporary use in intensive care units or heart disease strengthening treatment rooms.

## Safety management associated with the use of [^211^At]NaAt at radiation facilities

1. Management using logbooks
  When using [^211^At]NaAt, it is necessary to ensure radiation safety management, which includes proper usage methods to ensure radiation safety and storage of radioactive substances in a designated place. Therefore, items such as logbooks must always be kept and maintained [23].
2. Records regarding acceptance, use, storage, and disposal of [^211^At]NaAt (radiopharmaceutical usage log)
  The following are required to be entered in the usage logbook:
  (a) product standards, (b) date of arrival of goods, (c) date of use, (d) volume for use, (e) residual volume, (f) user, (g) patient name, (h) date of storage and disposal, and (i) radioactivity at storage and disposal.
  In addition, storage logbooks for medicines must be created. The storage capacity of the facility must be checked so as not to exceed the maximum storage capacity for each nuclide.
3. Measurement and log of places where radiation hazards may occur
  Concerning measurement at the boundary of controlled areas, residential areas, and radiotherapy and radioisotope use rooms [outside of the wall of the use room, in the use room, storage room, disposal facility (storage and disposal room, and drainage facility)], the amount of radiation and contamination status by radioisotopes must be measured once before starting medical treatment and once every month (for designated places, the period should not exceed 6 months) after the start of the medical treatment. The results must be recorded and stored for at least 5 years. Moreover, the radiation dose must be measured for a 1-cm dose equivalent (ambient dose equivalent: *H* ^*^(10)) rate (70-μm dose equivalent (directional dose equivalent: *H* ^‘^(0.07)) rate in places where the 70-μm dose equivalent rate may exceed 10 times the 1-cm dose equivalent rate). The radiation dose and status of radioactive surface contamination must be measured with a radiation measuring device. However, if this is difficult, these values may also be evaluated by calculations.
4. Measurement recording and exposure dose calculation in radiological medical personnel
  The effective and equivalent doses for radiological medical personnel are evaluated for external and internal exposure doses, and they are calculated based on the results obtained by the Minister of Health, Labour and Welfare [15].
5. Ionizing Radiation Medical Examination Individual Cards
  The results of the ionizing radiation medical examination for workers continuously engaged in radiological medical examinations should be recorded in the Ionizing Radiation Medical Examination Individual Cards.
6. Recording of the released patients administered [^211^At]NaAt
  Once release or returning home is permitted, the following must be recorded and kept for two years after release:
  - Administered dose, release date and time, and dose rate at release.
  - Notes and guidance for mothers with nursing infants.

## Radiation measurement

1. Measurement of dose (radioactivity).
  The measurement of the radioactivity of ^211^At will be carried out using a well-type ionization chamber called a dose calibrator or curie meter. The measurement method is the same as the conventional methods used for radioactive diagnostic drugs. ^211^At enclosed in a specified container (vial) is placed at the measurement position of a well-type ionization chamber using a jig for measurement. As ^211^At is a nuclide that has not been used to date, the well-type ionization chamber may not be calibrated for it (no calibration constant or dial setting for ^211^At). For first measurements, the measuring device must be calibrated in advance for ^211^At. Otherwise, the manufacturer must be contacted to set the calibration constant or dial setting.
2. Dose measurement at the place of use.
  When using radioisotopes for medical treatment, the air dose in the controlled area, the boundary of the controlled area, the boundary of the site, residential areas, etc., or the radiation dose at release of the patient and individual exposure dose of workers must be measured regularly or when necessary. ^211^At radiation control dosimetry is performed using gamma rays. The air dose is measured using a 1-cm dose equivalent *H* ^*^(10) as the ambient dose, and the exposure dose is measured using a device calibrated with the 1-cm dose equivalent *H*_p_(10) as the individual dose equivalent.

For the air dose measuring device, a survey meter with a gamma-ray detector such as an ionization chamber or a NaI (Tl) scintillation detector as the detection unit can be used. Ionization chambers are suitable for measurements in places with relatively high dose rates, such as places of use. A highly sensitive NaI (Tl) scintillation survey meter is effective in low-dose areas such as controlled area boundaries and site boundaries. Furthermore, the cumulative dose during the period (1 week or 3 months) should be calculated appropriately based on the dose rate measured using the above survey meters (generally, the indicated in [μSv/h]); however, a device capable of measuring the cumulative dose (e.g., passive dosimeter) may be used.

Some personal dosimeters directly display the exposure dose, whereas others calculate it with a reading device worn for a certain period of time. For the passive dosimeter, the exposure dose is generally read by a personal dosimetry service organization. As those that directly display the exposure dose are placed in the pocket, they are also referred to as direct-reading pocket dosimeters, and recently, those that use semiconductors such as Si have been frequently used. Film badges were the mainstream passive dosimeters. Currently, fluorescent glass dosimeters and optically stimulated luminescence dosimeters are mainly used.

## Education and training

Knowledge on medical safety related to this treatment and safe handling of radiation must be acquired. Education and training based on this manual at each medical institution must be conducted on the following:

- Laws, reports, and release criteria related to radiation hazard prevention.
- Chemical and physical properties of [^211^At]NaAt and radiological protection.
- Exposure prevention of medical staff and instructions for patients and their families.
- Radiation measurement and safety management of radioactive waste.
  Physicians who have acquired specialized knowledge through education and training conducted at the hospital can be administrators of the therapy. In that case, it is ideal for the physician, to be appointed as the manager or investigator at the hospital to which they are affiliated.
  In addition, the education and training conducted in the hospital must be recorded. Implementation records must be kept for at least 2 years.

## Radiological protection measures related to the handling of [^211^At]NaAt

1. Preparation of protective equipment
  - Protective glasses (required): Anticipate the possibility of the product directly contaminating the eyes in the process of handling.
  - Protective gloves (required): To prevent direct contamination of fingers/hands when handling [^211^At]NaAt.
  - Water-absorbent polyethylene filter paper: Polyethylene filter paper absorbs water-containing radioactive substances and prevents the spread of pollution. Cover the inside of the potentially contaminated biosafety cabinets, the work surface around it, and lead blocks using polyethylene filter paper.
  - Tweezers: Attaching a silicon tube to the tip of tweezers prevents slips and makes it easy to grab the vial.
  - Appropriate size vat: If a water-absorbent polyethylene filter paper is placed on an appropriate size stainless steel vat and spread on it, even if radioactive liquid spills during operation, the radioactive contamination can be retained in the vat, which helps prevent the spread of the contamination.
2. Fundamentals of handling of radioactive substances.

Due circumspection must be practiced when handling radiopharmaceuticals, which are unsealed radioisotopes, because aside from external exposure, internal exposure also occurs when these radioisotopes enter the body. Furthermore, unlike sealed radioisotopes, radiopharmaceuticals are often handled at close range. Moreover, it should also be considered that patients administered radiopharmaceuticals are also sources of radiation exposure. Therefore, when handling [^211^At]NaAt, exposure must be reduced by reducing working time, keeping a safe distance from the radiation sources, and installing shields (three principles for external exposure protection).

1. Implementation of cold run (practice of operation to handle [^211^At]NaAt)
  The act of performing the procedure involving radioisotopes (RI) without using actual RI is called cold run.
  - The work procedure can be checked and understood by repeating cold runs and practicing and gaining the necessary skills
  - The preparation of necessary equipment and protective elements can be checked.
  - Mistakes can be reduced and speed can be increased in the operation involving actual radioactive substances.
  In other words, it is possible to speed up the handling of the radiation source (reduce time) and minimize operational mistakes.
2. Precautions in the controlled area
  Precautions for entering and exiting RI-controlled areas and laboratories should be posted near the entrances and exits in compliance with the Medical Care Act. Therefore, it is necessary for radiological medical personnel involved in radiation work to thoroughly implement this precaution. The main precautions are listed below:
  - An entry log must be recorded.
  - Radiation clinic workers should change to slippers, athletic shoes, biosafety shoes, etc., which will be exclusively used in the controlled area.
  - Radiological medical personnel should change into work clothes that will be exclusively used in the controlled area.
  - A personal exposure dosimeter such as a pocket dosimeter must be worn on the chest for men and on the abdomen for women.
  - Check that the ventilation system of the exhaust equipment is in the operation room.
  - Protective glasses and gloves must be worn when handling radiopharmaceuticals.
  - After use, disposed radiopharmaceuticals and radioactive substances must be moved to the storage and disposal room immediately.
  - After use, the room must be inspected for radioactive contamination, and if contaminated, decontamination must be performed immediately.
  - Hands must be washed with detergent and running water.
  - Hands, feet, cuffs, clothing surface, footwear, etc., must be inspected for contamination.
  - If there is no contamination, clothes must be changed. If contaminated, perform decontamination following the instructions of the radiation manager.
  - An exit log must be recorded.
  - Read and record the value in the personal exposure dosimeter.
3. Handling of [^211^At]NaAt.
  Dispensing [^211^At]NaAt: Generally, when the dose of [^211^At]NaAt needs to be reduced, it must be dispensed in a biosafety cabinet. Check that the biosafety cabinet is working properly. In addition, the floor around the biosafety cabinet must be covered with polyethylene filter paper to allow easy decontamination. If necessary, the work surfaces inside the cabinet, the back, front, and sides must also be covered with polyethylene filter paper. Furthermore, when handling radiopharmaceuticals, a shield such as a lead plate or a block must be used to reduce the exposure to radiological medical personnel.

Administration of [^211^At]NaAt: This drug should be slowly administered intravenously. When administering [^211^At]NaAt, measures must be taken to control exposure and contamination in radiological medical personnel.

Procedure for handling [^211^At]NaAt and waste disposal after administration: Protective glasses must be worn when handling this drug. In addition, protective equipment such as lab coats and gloves must be worn. Handle [^211^At]NaAt in a stainless steel vat covered with water-absorbent polyethylene filter paper. The same applies when treating contaminated objects. In the unlikely event that the surface of the skin (e.g., face) or the eyes have been contaminated with this drug, immediately wash thoroughly with detergent and running water.

Radiological medical personnel should not leave the site or walk around while performing radiological work such as preparation of pharmaceuticals. Immediately after completing work, waste should be separated and stored for disposal.

Contamination inspection and decontamination of rooms (walls, floors, etc.) in which [^211^At]NaAt was used: the presence or absence of contamination by [^211^At]NaAt should be confirmed by measuring radioactivity in the biosafety cabinet using a radiation measuring device along the flow line where this drug was used.

As ^211^At emits α-rays and X-rays, it is important to use a radiation measuring device that effectively detects ^211^At surface contaminations. Simultaneous preparation and dispensing of other medical nuclides in the room may lead to misadministration and should be avoided to ensure medical safety.

Generally, it is ideal to measure alpha rays using a ZnS (Ag) scintillation survey meter to detect radioactivity on the workbench or floor surface contaminated by ^211^At. However, a NaI (Tl) scintillation survey meter having a sensitivity to 80 keV Po-K X-rays and a GM survey meter with sensitivity to 80 keV Po-K X-rays or 12.4 keV Po-L X-rays (see Table 1) can also be used simultaneously.

If the workbench or floor is contaminated, immediate decontamination must be performed. If contamination is found relatively early, the general protocol is to absorb it with a paper towel and gradually decontaminate the area with water, a neutral detergent, or a chelating reagent such as citric acid. When decontaminating, pay attention to cracks and pinholes in the gloves to prevent secondary contamination of the body. If complete decontamination is not possible, using a marker delineate the scope of contamination, measured dose, and the date of contamination to demarcate the contaminated area. In addition, preventing the spread of contamination by keeping people away from the areas of concern is also an appropriate radiation exposure- and pollution-prevention measure.

## Exposure of medical personnel (external and internal exposure)

Managers of healthcare facilities are responsible for preventing exposure of medical personnel in accordance with Articles 30–18 and 30–27 of the Medical Care Act Implementing Regulations, Items 1 to 2 related to No. 5: limitations in the HPB 0315, No. 4 Notice and 1 to 5 in No. 6: Calculation of dose.

The maximum single dose in this treatment is 1 [GBq], and the external exposure dose of medical personnel is calculated as shown in Table 7 based working time and distance from the radiation source.

**Table 7:** External exposure dose of healthcare workers

Stage of work	Effective dose (per case)			Skin dose *(per case)			Dose limit	
	Working time (min)	Distance (cm)	Exposure dose (mSv)	Working time (min)	Distance (cm)	Exposure dose (mSv)	Effective dose limit (whole body)	Equivalent dose limit (skin)
Preparation	10	50	0.0043	10	10	0.107	Radiological professionals: 50 mSv/year, 100 mSv/5 years Women who can be pregnant: 5 mSv/3 months	500 mSv/year
Administration	5	50	0.0021	5	5	0.215		

*Reference value using the effective dose rate constant. The equivalent dose to the skin should be measured at a dose equivalent of 70 µm

The effective dose [mSv/week], *E,* due to internal exposure of workers per week is calculated using the following formula based on Notice of the Ministry of Health, Labour and Welfare [15, 24].

$$E\, = \,e\, \times \,I.$$

where*I* is the quantity [Bq] of medical radioisotopes inhaled per week, which is calculated as:

$$I\, = \,{1}.{2}\, \times \,{1}0^{{6}} \, \times \,C\, \times \,t.$$

1.2× 10^6^: Volume of air inhaled by an adult per hour in [cm^3^/h.]

*C*: Average radioactivity concentration in the air per week in [Bq/cm^3^.]

*t*: Working hours [/week].

*C* = *A* × scattering rate × number of days used per week/[*V* × 10^6^ × 8 [h] × number of operational days of exhaust equipment per week]

*A*: Maximum daily usage quantity [Bq].

*V*: Indoor displacement [m^3^/h] must be operational for 8 [h/day] at a displacement of*V* [m^3^/h].

In the case of [^211^At]NaAt, A: 1.0 [GBq]; scattering rate: 0.001; daily indoor displacement: 560 [m^3^/h] × 8 [h]; weekly use: 1 [day]; weekly exhaust equipment operational days: 5 [day]; working time: 10 [min] (0.167 [h]); and*e* (effective dose coefficient when ^211^At is inhaled): 2.7 × 10^–5^ [mSv/Bq]. The effective dose*E* [mSv] because of internal exposure per week is determined as follows:

$$C = {1}000 \times {1}0^{{6}} \times 0.00{1} \times {1}/\left( {{56}0 \times {1}0^{{6}} \times {8} \times {5}} \right) = {4}.{47} \times {1}0^{{ - {5}}} \left[ {{\text{Bq}}/{\text{cm}}^{{3}} } \right]$$

$$I\, = \,{1}.{2}\, \times \,{1}0^{{6}} \, \times \,C\, \times \,0.{167}\, \times \,{1}\, = \,{8}.{96 }[{\text{Bq}}].$$

$$E\, = \,e\, \times \,I\, = \,{2}.{7}\, \times \,{1}0^{{{-}{5}}} \, \times \,{8}.{96}\, = \,{2}.{4}\, \times \,{1}0^{{{-}{4}}} [{\text{mSv}}].$$

## Precautions for medical personnel

Medical personnel involved in nuclear medicine treatment with [^211^At]NaAt must fully understand this manual as well as the pharmacokinetics of this drug, and then, explain the above-mentioned principles regarding radiological protection to patients and their families in a clear manner. In addition, physicians with specialized knowledge of this treatment should provide appropriate education and training to medical personnel and strive to enhance cooperation in the medical institution. If urgent medical treatment is required, appropriate medical treatment may be prioritized over the above-mentioned compliance items regarding radiological protection to save the lives of patients.

Those engaged in patient care must pay attention to the following within one week of administration.

1. Wear disposable rubber gloves when there is a possibility of contact with the patient’s urine, feces, or blood, or when handling clothing contaminated by these.
2. If there is contact with the patient’s excrement or blood, immediately wash the contaminated parts thoroughly with soap and running water.
3. Wash clothes contaminated with patient excrement or blood separately from other people’s clothes.

## Disposal of medical radioactive contaminants (contaminated with ^211^At)

Objects contaminated with [^211^At]NaAt fall under “medical radioactive contaminants” as stipulated in Article 30–11 of the Medical Act Implementing Regulations. Medical radioactive contaminants should be stored and disposed of at a “disposal facility” in hospitals, based on the provisions of Article 30–11 of the same Act. Furthermore, the corresponding contaminated objects can be inquired of the person entrusted with the disposal of the medical radioisotope or objects contaminated with the radioisotope as stipulated in Article 30–14-2, Clause 1 of the Act.

When handling human excrement in diapers and urine bags and items contaminated with blood, please refer to Handling Diapers of Patients administered Radiopharmaceuticals (Guidelines for Medical Professionals Practicing Nuclear Medicine) and “Handling manual for diapers of patients administered radiopharmaceuticals” [17].
